# Supplementary material for: Therapeutic Potential of a Senolytic Approach in a Murine Model of Chronic GVHD
Source: Biology (Basel). 2023 Apr 25;12(5):647. doi: 10.3390/biology12050647 (PMC10215844; doi:10.3390/biology12050647)
Supplement: Supplementary file 1 [file biology-12-00647-s001.zip › biology-2300055-supplementary.pdf]

Supplementary Figure S1A

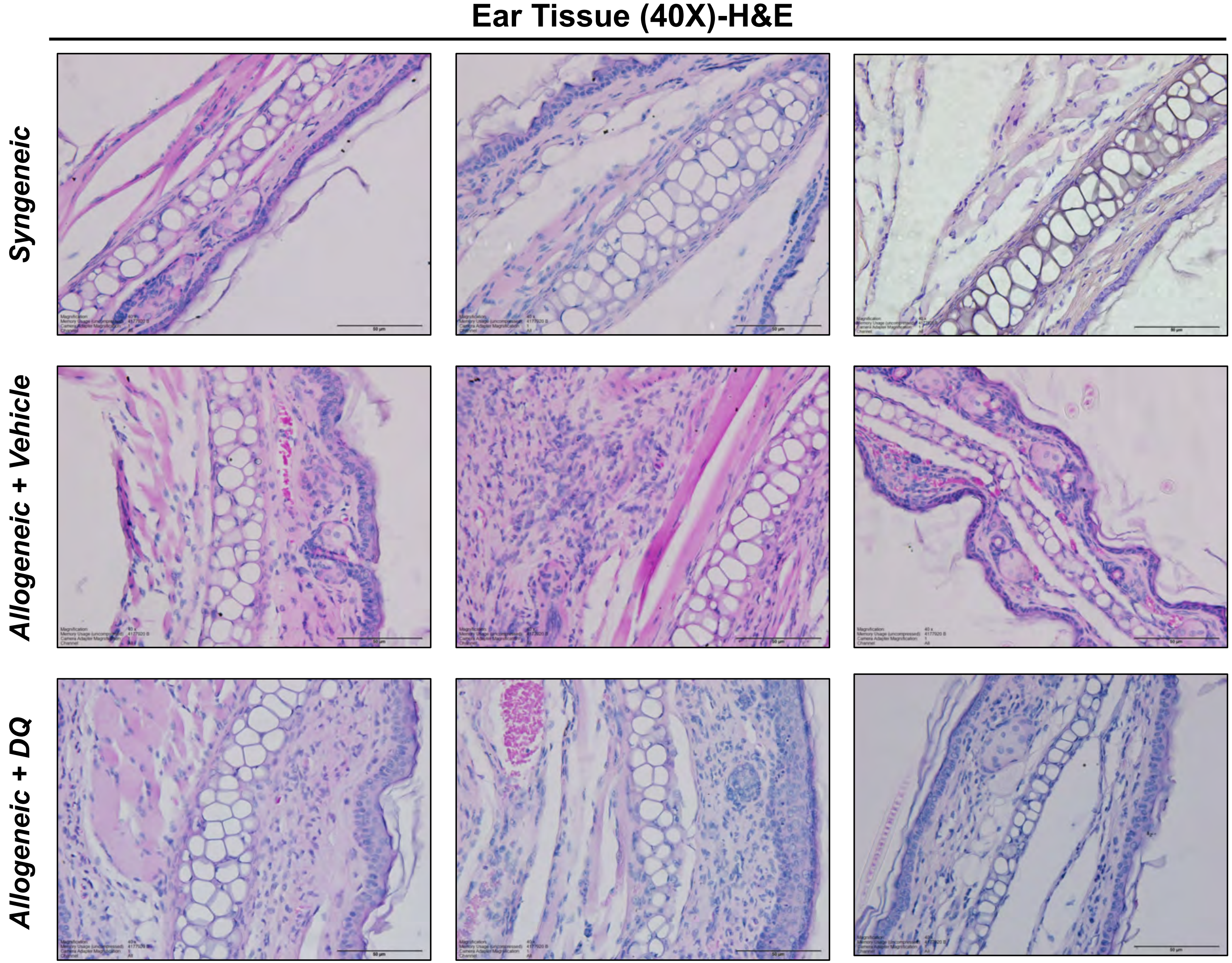

Supplementary Figure S1B

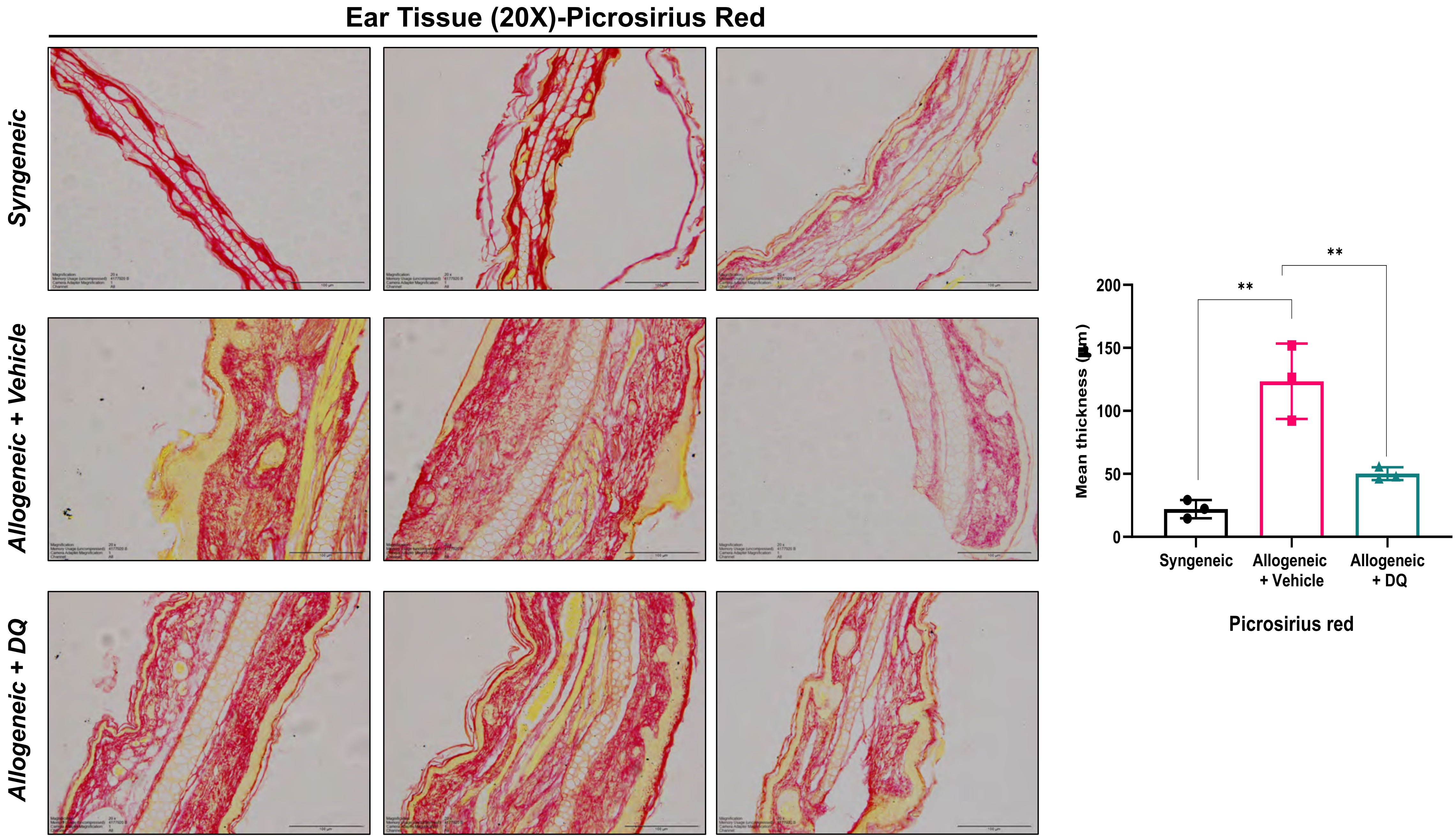

Supplementary Figure S1C

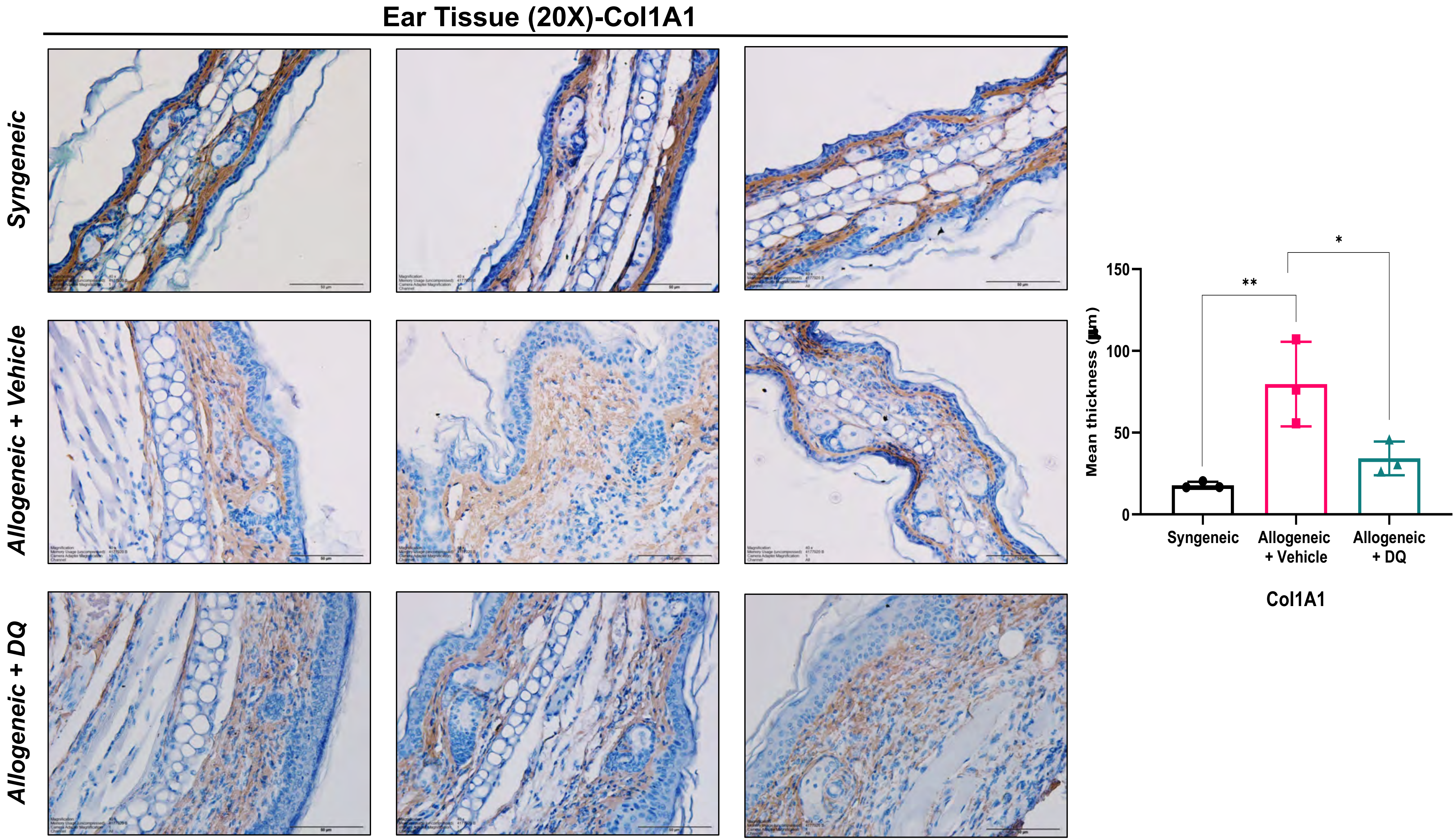

Supplementary Figure S1D

Ear Tissue (40X)-αSMA

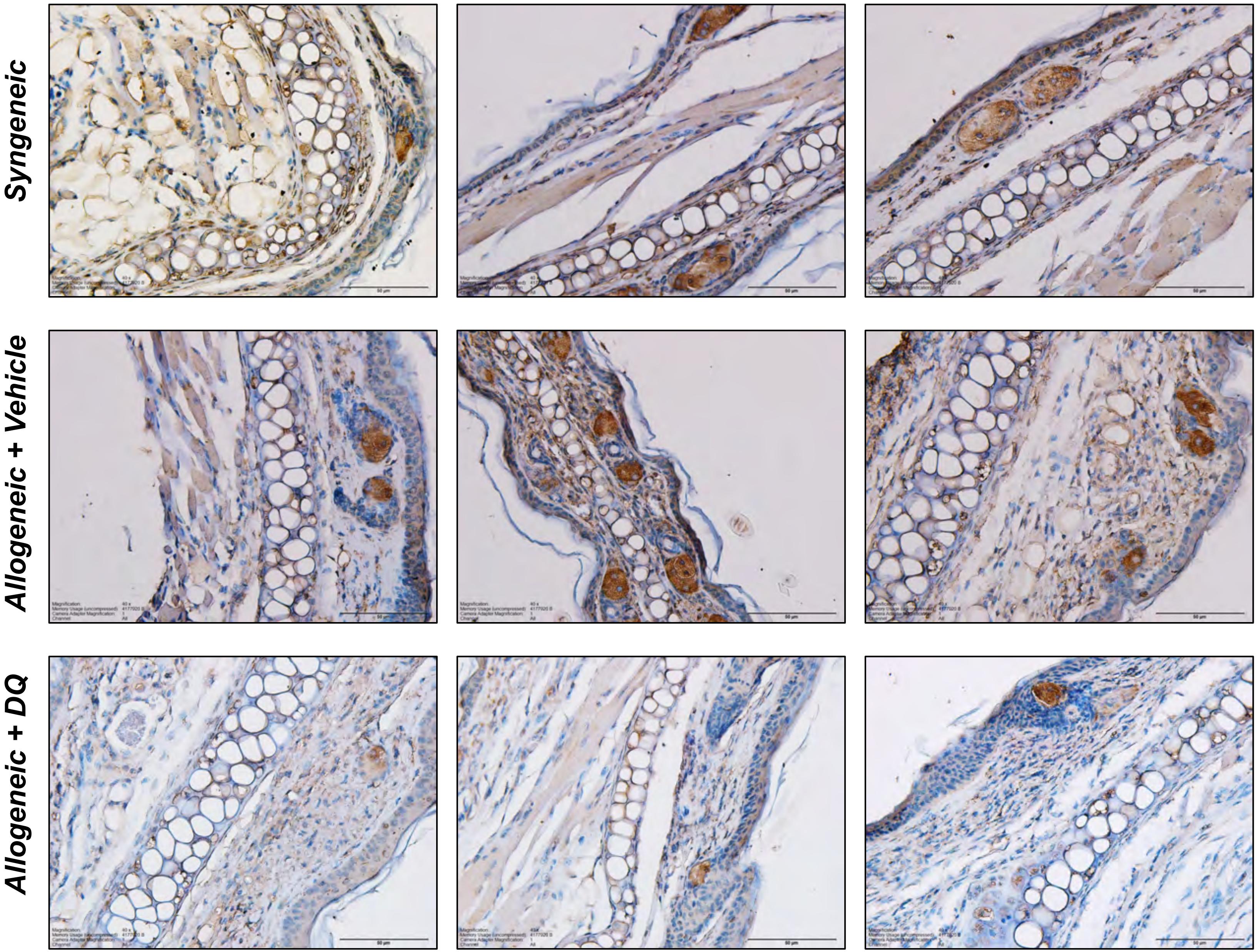

Supplementary Figure S2A

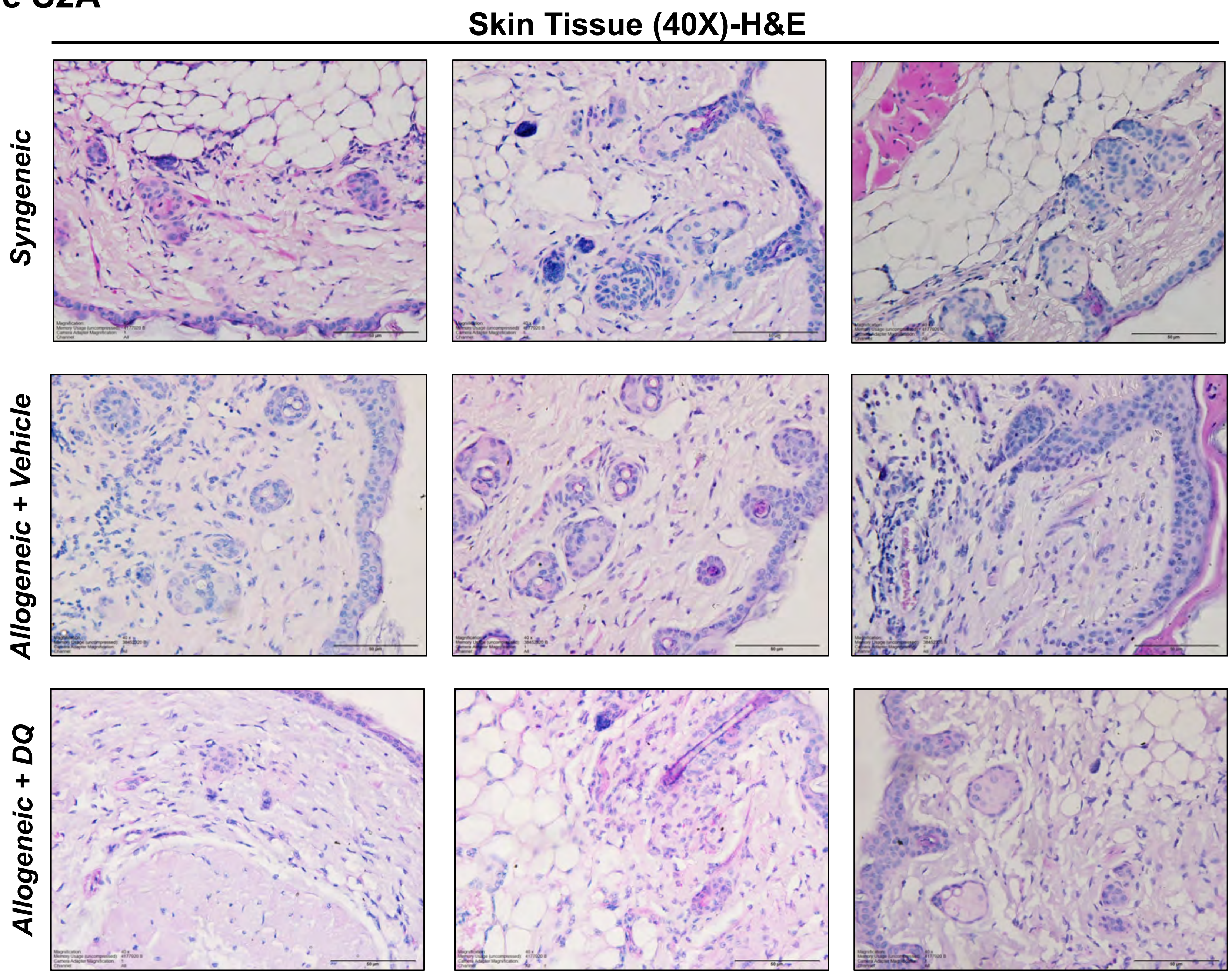

Supplementary Figure S2B

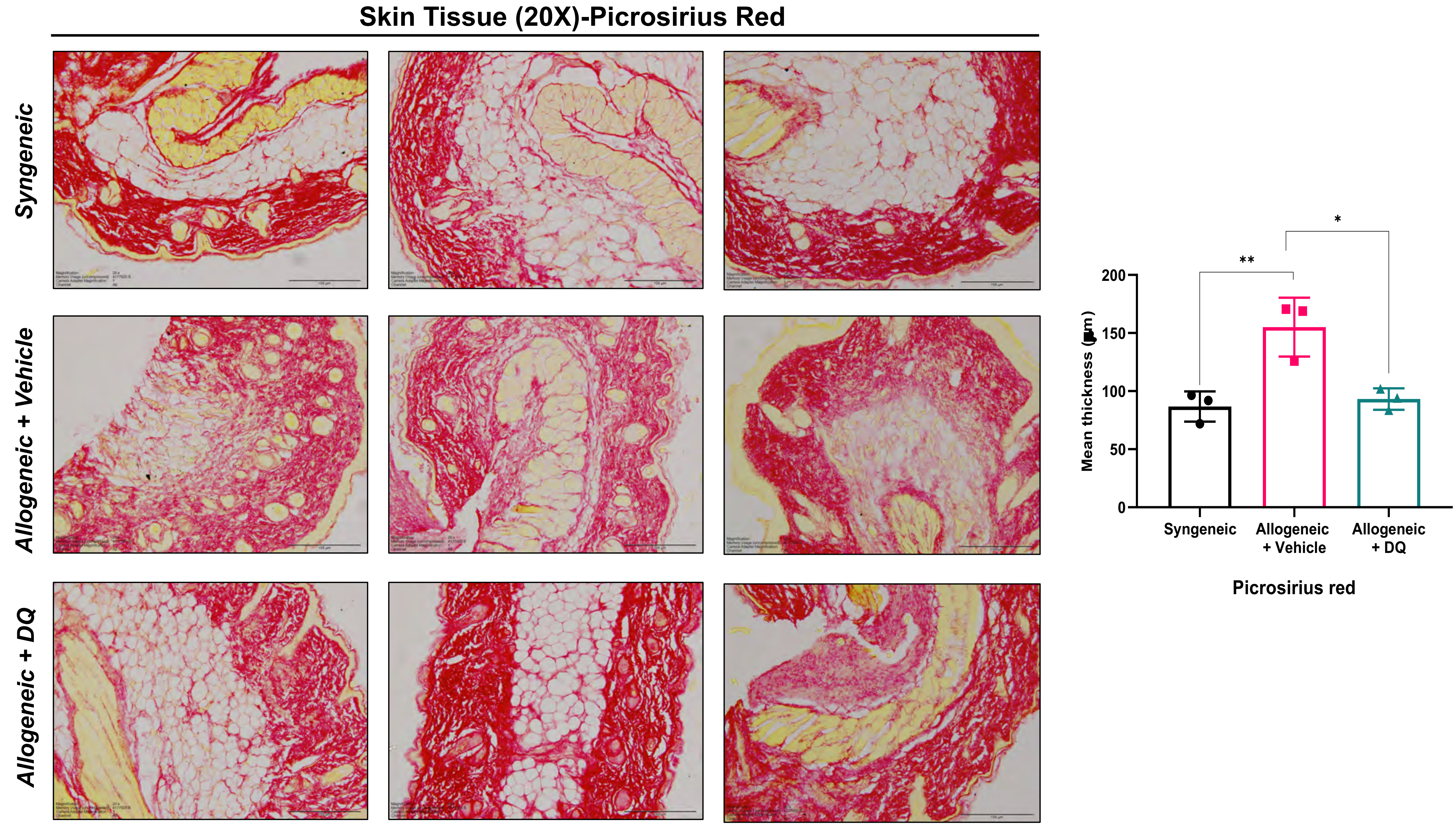

Supplementary Figure S2C

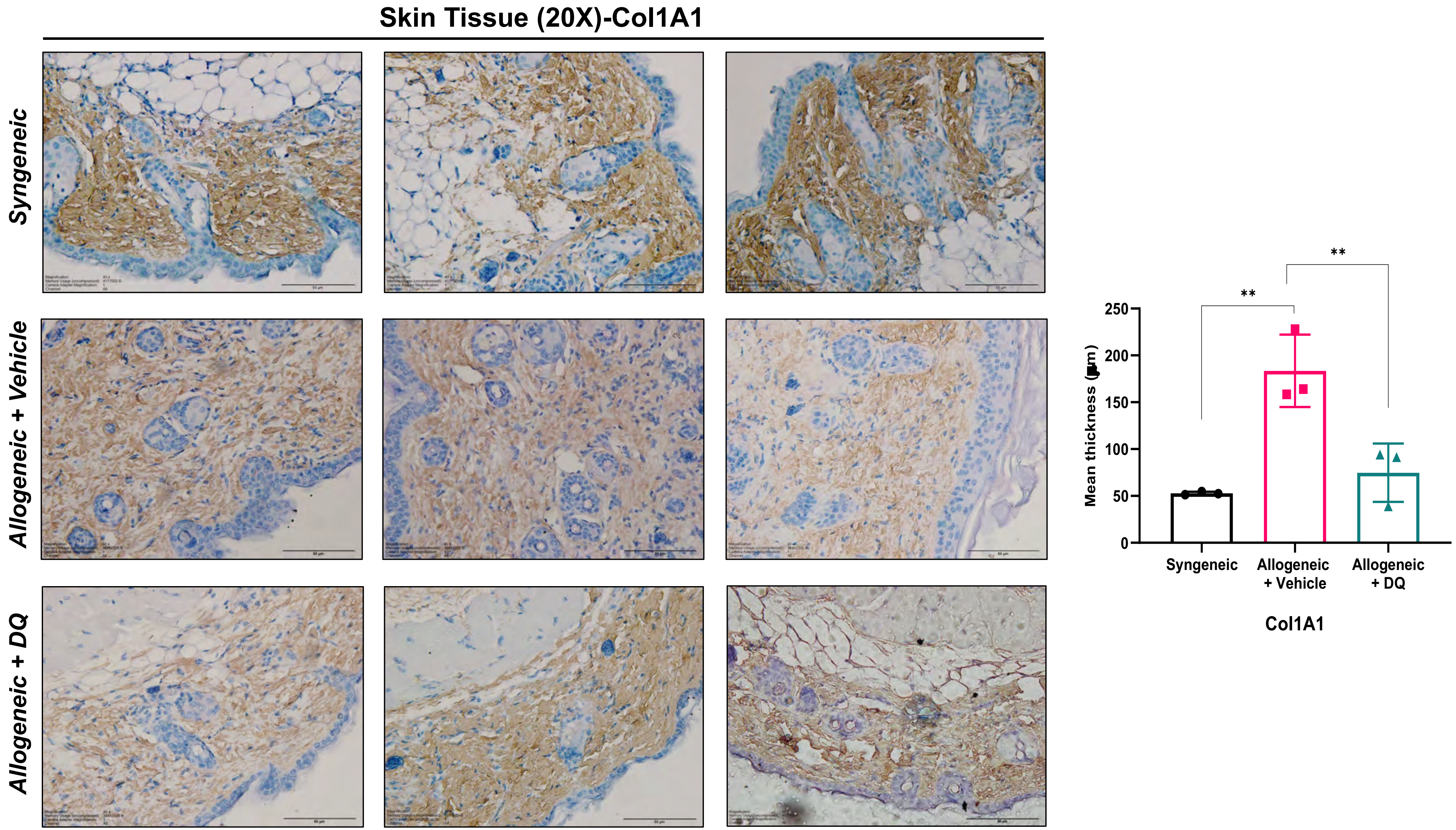

Supplementary Figure S2D

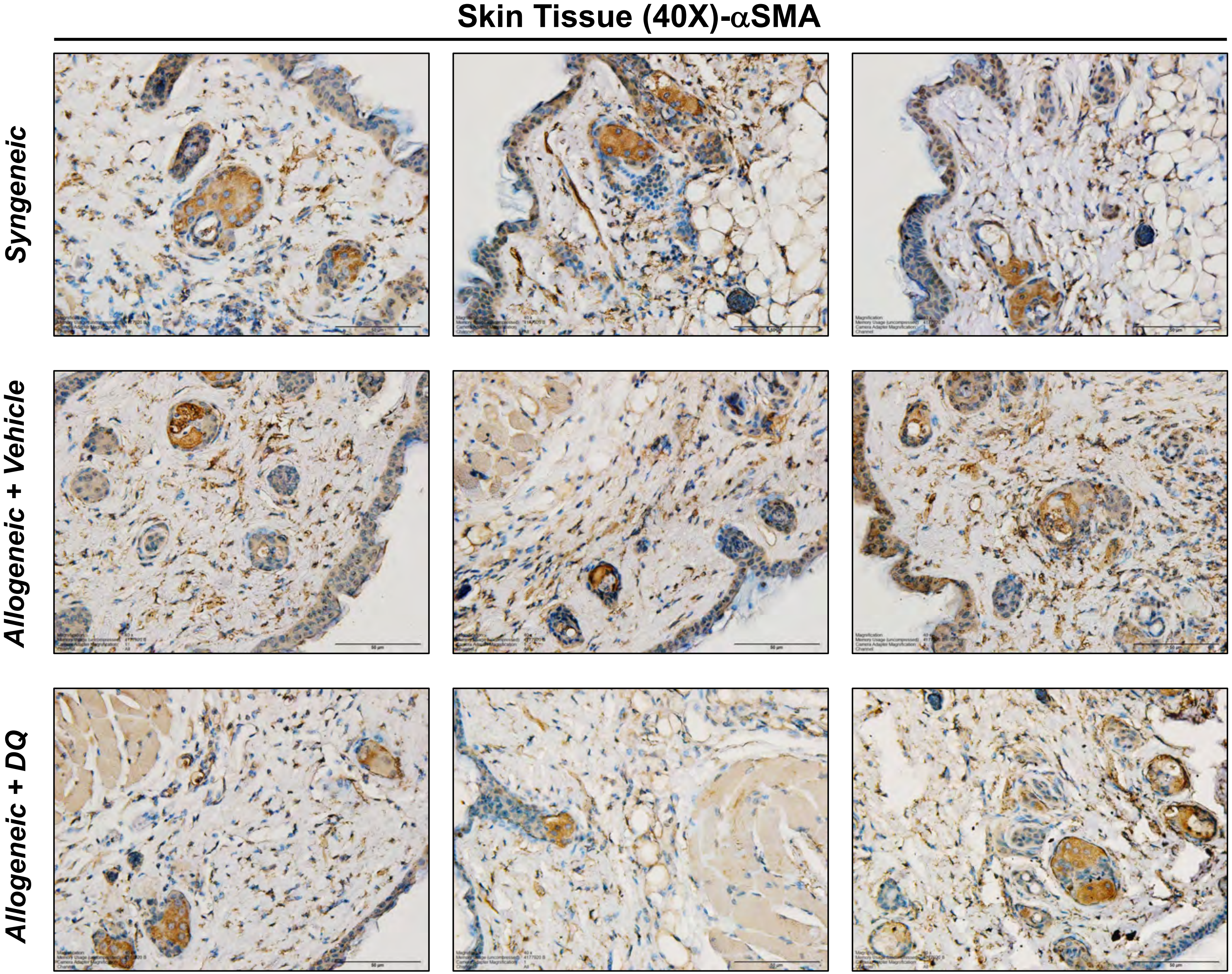

Supplementary Figure S2E

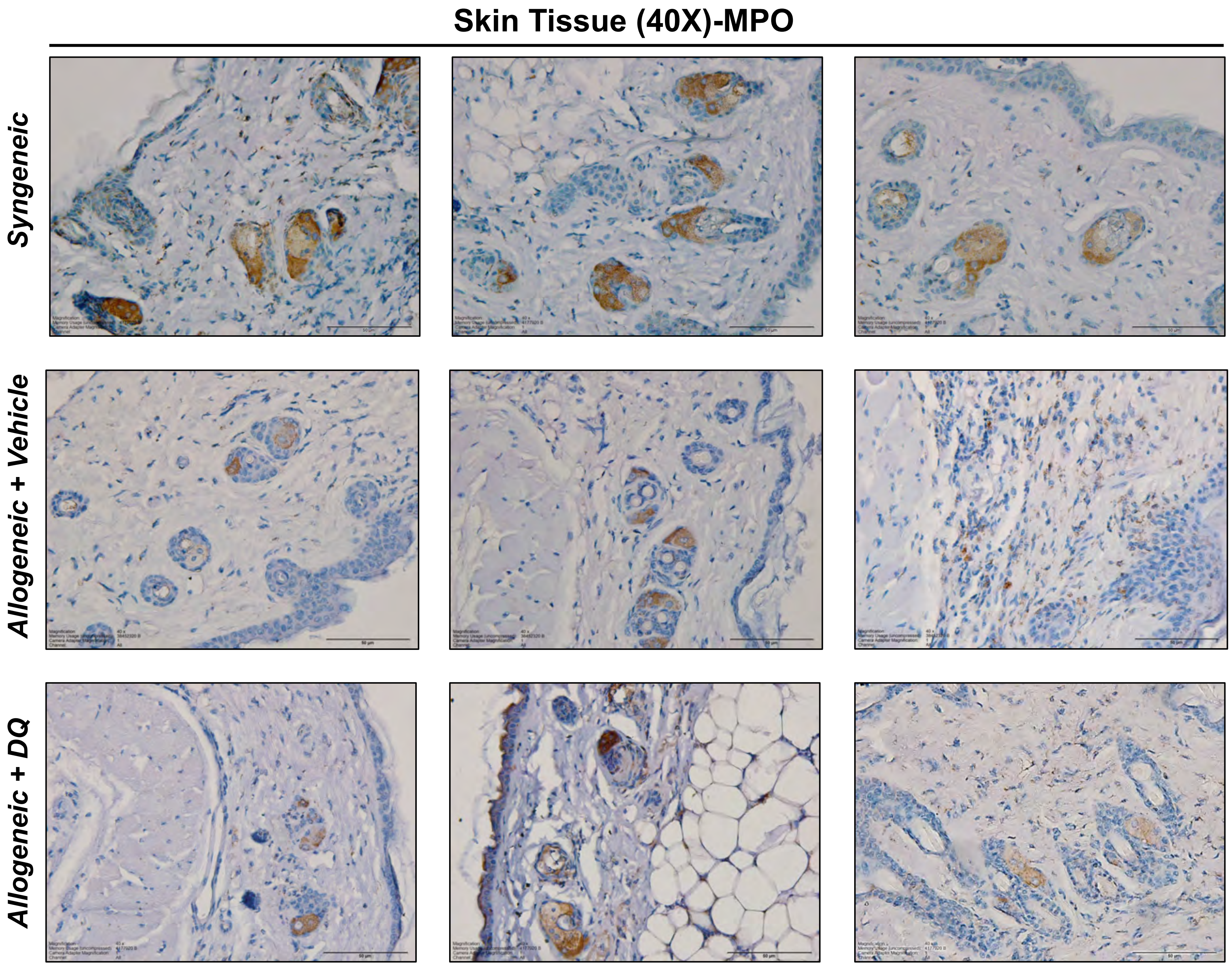

Supplementary Figure S2F

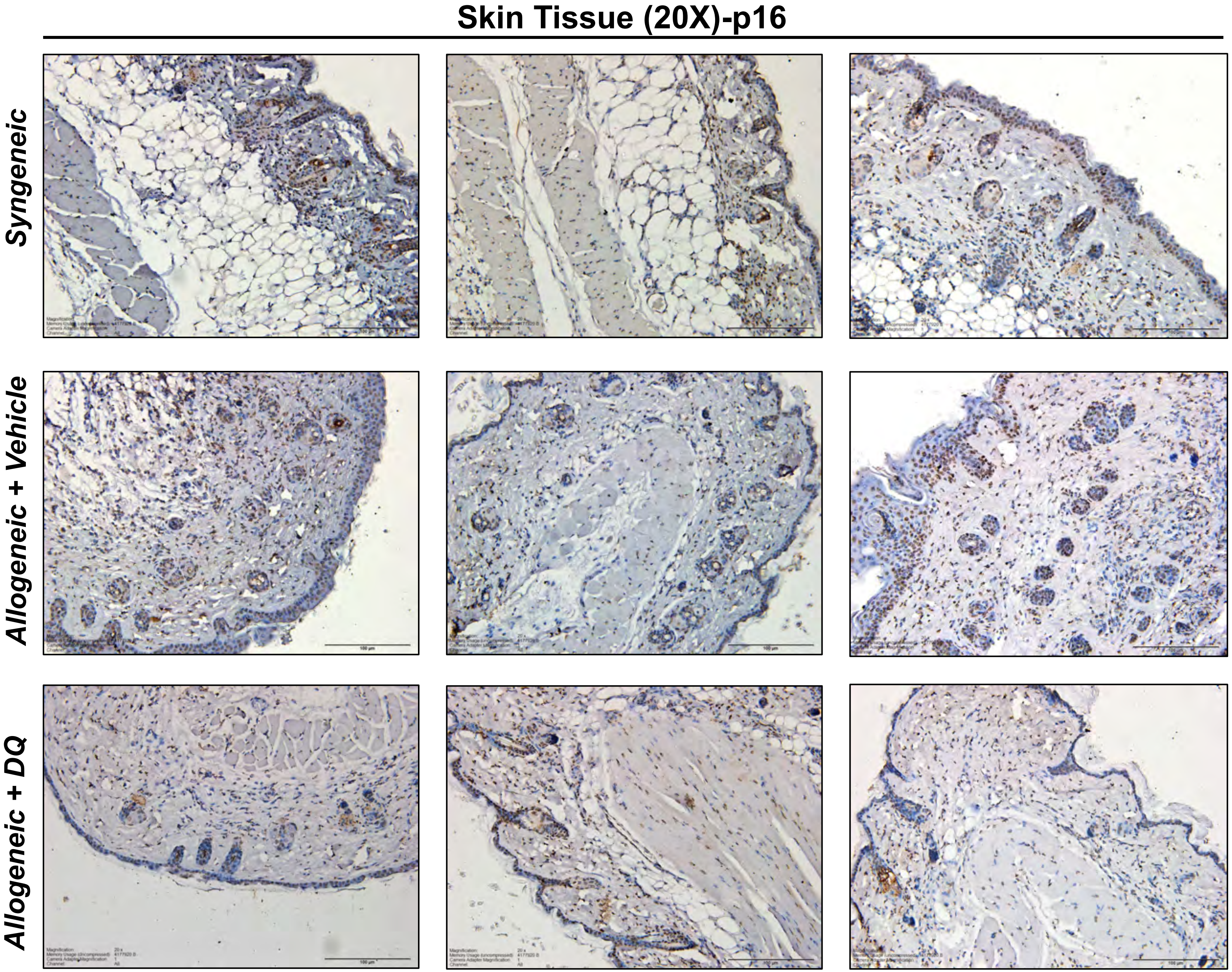

Supplementary Figure S3

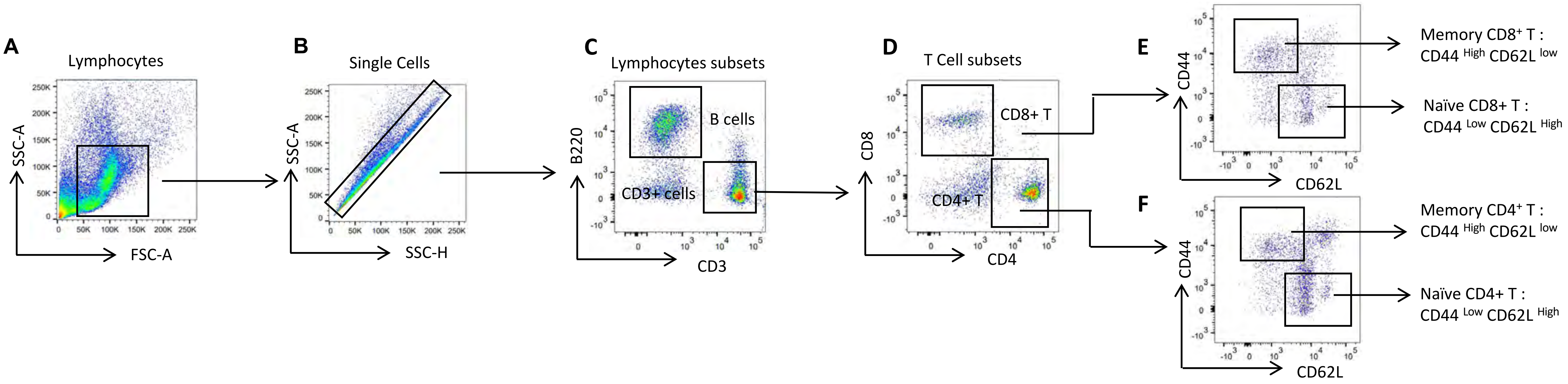

Supplementary Figure S4

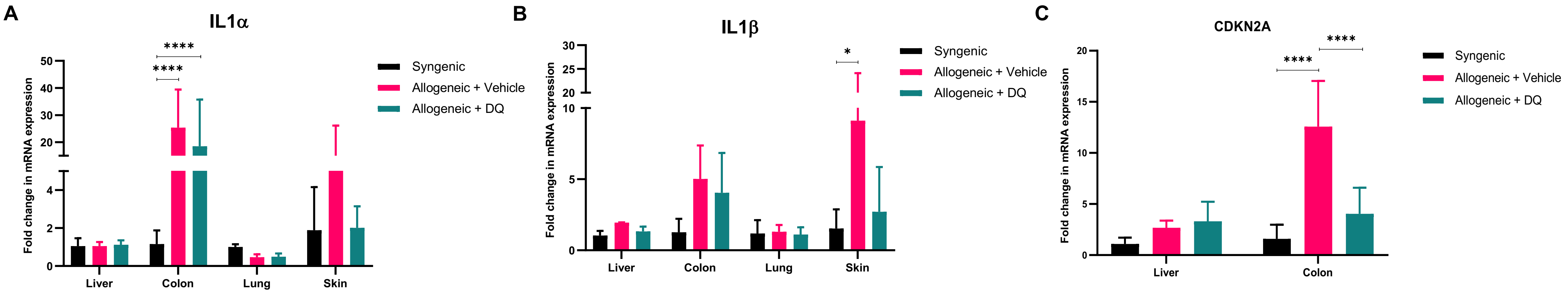

Supplementary figures

Supplementary Figure S1: DQ treatment ameliorates cGvHD associated fibrosis in ear tissues

Representative images of ear tissue from syngeneic recipients, allogeneic recipients and allogeneic recipients after DQ treatment were stained with (A) H&E (40X) and (B) picrosirius red (20X). Average width of picrosirius red stained section was calculated using imageJ software. One-way ANOVA analysis was used for statistical significance (** = p<0.01). (C) Ear tissue sections (40X) were stained with anti-col1A1. Average width of collagen-stained section was calculated using imageJ software. One-way ANOVA analysis was used for statistical significance (* = p<0.05, ** = p<0.01). (D) Ear tissue sections (40X) from mice in the three groups were stained for α-SMA.

Supplementary Figure S2: DQ treatment ameliorates cGvHD associated fibrosis in skin tissues

(A) Representative images of hematoxylin and eosin stain (40X) and (B) picrosirius red (20X) stain opf skin tissue. Average width of picrosirius red stained section was calculated using imageJ software. One-way ANOVA analysis was used for statistical significance (* = p<0.05, ** = p<0.01). (C) Image (40X) of tissues stained with col1A1 antibody. Average width of collagen-stained section was calculated using imageJ software. One-way ANOVA analysis was used for statistical significance (** = p<0.01). (D) Images of skin tissue sections from syngeneic and allogeneic graft recipients stained with anti SMA (40X), (E) anti MPO (40X) and (F) anti p16 (20X)

Supplementary Figure S3 : Gating strategies used for flow cytometry analysis of splenic T and B cells (sub)populations : (A) From initial population , FSC ^{low} and SSc ^{low} population was gated excluding the debris (<50K). (B) Single cells were then considered. (C) B cells were defined as $CD3^{-}$ B220 $^{+}$ and T cells as $CD3^{+}$ $B220^{-}$. (D) Within the global T $CD3^{+}$ cell population $CD4^{+}$ $CD8^{-}$ and $CD4^{-}$ $CD8^{+}$ were identified. Naïve and memory phenotypes were defined on the level of CD44 and CD62L expression as defined in the figure (E-F). Analysis was performed using FlowJo v10.7 software (BD Biosciences, San Jose, California, USA)

Supplementary Figure S 4: DQ treatment modulates SASP response and cytokines in cGvHD pathogenesis: (A-B) qRT-PCR analyses of cytokines IL-1α and IL-1β involved in SASP determine tissue specific activity of DQ in alleviating cGvHD. Two-way ANOVA analysis was used for statistical significance (* = p<0.05, **** = p<0.0001). (C) Induction of senescence associated *CDKN2a* mRNA in cGvHD is significantly alleviated by DQ in the colon. Two-way ANOVA analysis was used for statistical significance (**** = p<0.0001)
